# 

**Published:** 1853-02

**Authors:** 


VOL. I. NEW SERIES. No. 10.

### THE NORTH-WESTERN MEDICAL AND SURGICAL JOURNAL.

EDITED BY

W. B. HERRICK, M.D.,

PROFESSOR OF ANATOMY AND PHYSIOLOGY IN RUSH MEDICAL COLLEGE; SURGEON TO THE U.S. MARINE HOSPITAL, ONE OF THE SURGEONS OF THE ILLINOIS GENERAL HOSPITAL, ETC.

ASSISTED BY

H. A. JOHNSON, M.D.

PUBLISHED MONTHLY,

AT TWO DOLLARS PER ANNUM.

FEBRUARY, 1853.

CHICAGO:

PUBLISHED BY BALLANTYNE & CO., PRINTERS & BOOKBINDERS.

NEW POST-OFFICE BUILDINGS, COR. OF CLARK AND RANDOLPH-STS.,
OPPOSITE THE SHERMAN HOUSE.

1853.

VOL. IX. WHOLE SERIES. No. 10.

